# Effects of Seedling Substrate and Hydroponic Versus Aquaponic Nutrient Solution on Growth, Nutrient Uptake, and Eco-Physiological Response of Lemon Basil (*Ocimum × citriodorum*)

**DOI:** 10.3390/plants14131929

**Published:** 2025-06-23

**Authors:** Linda Signorini, Giuseppe Carlo Modarelli, Prospero Di Pierro, Antonio Luca Langellotti, Chiara Cirillo, Stefania De Pascale, Paolo Masi

**Affiliations:** 1 Department of Agricultural Sciences, University of Naples Federico II, Piazza Carlo di Borbone 1, 80055 Portici, Italy; linda.signorini@unina.it (L.S.); prospero.dipierro@unina.it (P.D.P.); depascal@unina.it (S.D.P.); pmasi@unina.it (P.M.); 2 Leibniz Institute of Vegetables and Ornamental Crops (IGZ), 14979 Großbeeren, Germany; 3 Center for Innovation and Development of the Food Industry (CAISIAL), University of Naples Federico II, Via Università 100, 80055 Portici, Italy; langello@unina.it

**Keywords:** lemon basil (*Ocimum × citriodorum*), soilless cultivation, aquaponics, flavonoids, coconut fiber

## Abstract

Lemon basil (*Ocimum × citriodorum*) is a highly valued aromatic plant renowned for its distinct citrus aroma. This study aimed to evaluate sustainable substrates and cultivation systems for its production. Two complementary and sequential experiments were conducted: an initial experiment designed to compare coconut fiber mixed in varying proportions with perlite to rock wool, evaluating their effectiveness during germination and early growth (experiment 1), and a subsequent experiment aimed at assessing plant performance in a decoupled aquaponic system relative to hydroponics utilizing the best-performing coconut fiber-perlite mixture from the first phase along with rock wool as substrates (experiment 2). The substrate with 70% coconut fiber and 30% perlite (F70:P30) significantly improved seed germination, leaf number, and total leaf area of seedlings. The decoupled aquaponic cultivation system resulted in a 52.5% increase in flavonoid content, accompanied by higher calcium and magnesium uptake in stems and roots compared to hydroponics. These findings clearly underscore the potential of coconut fiber substrates mixed with perlite as sustainable alternatives to rock wool, reducing environmental impact, disposal costs, and health risks. Similarly, aquaponic cultivation emerges as a valuable strategy for sustainable lemon basil (*Ocimum × citriodorum*) production, offering comparable yields to hydroponics while improving plant nutritional and phytochemical quality through beneficial plant-microbe interactions. These results provide practical evidence supporting the adoption of environmentally friendly substrates and cultivation practices, thus contributing significantly toward sustainable intensive vegetable production systems.

## 1. Introduction

Basil (*Ocimum basilicum* L.) is a highly appreciated aromatic herb worldwide, renowned for its culinary versatility and distinctive fragrance [1]. Among its varieties, lemon basil (*Ocimum × citriodorum*), a hybrid between basil (*Ocimum basilicum*) and African basil (*Ocimum americanum*), has recently gained increasing global popularity due to its unique, fresh citrus aroma which enhances various dishes, particularly seafood recipes, by adding freshness and vibrancy [1,2].

Aromatic herbs and leafy vegetables are widely cultivated worldwide. In Italy, one of the main production areas is the “Piana del Sele” in Campania, an intensive agricultural area spanning around 250 km^2^ that has become a major hub for greenhouse vegetable production, especially fresh, ready-to-eat (“fourth range”) leafy vegetables exported throughout Europe [3]. Over the past two decades, the rapid expansion and industrialization of agriculture in this area have led to significant environmental challenges, including soil degradation, salinization, soil compaction, coastal erosion, and the accumulation of potentially toxic substances due to the intensive use of agrochemicals [4,5].

Recently, many farms in the area have been confronted with increased contamination risks, particularly related to heavy metals such as cadmium and lead accumulating in leafy vegetables, posing threats to food safety, human health, and economic sustainability [6].

To address these challenges, soilless cultivation systems, such as hydroponics or aquaponics, have emerged as a promising alternative to traditional soil cultivation.

These methods can mitigate environmental impacts by significantly reducing agrochemical use, shortening crop cycles, minimizing water and nutrient losses, and avoiding soil-related contaminant issues [7,8]. Hydroponics allows precise control of nutrient supply tailored to plant needs; however, it still heavily relies on chemical fertilizers, leading to environmental concerns and associated economic costs [8,9]. In contrast, aquaponics integrates aquaculture and plant cultivation within a closed-loop system, using nutrient-rich fish wastewater as fertilizer for plant growth. Beneficial microorganisms convert fish waste into bioavailable nutrients, creating a synergistic and sustainable nutrient cycle. This reduces chemical fertilizer inputs, optimizes resource use efficiency, and minimizes environmental pollution [9,10].

Another significant sustainability issue in greenhouse vegetable production involves substrate selection. Generally, rock wool is still the most widespread substrate used in soilless cultivation despite its environmental drawbacks, high disposal costs, and potential toxicity risks during handling [11]. Therefore, identifying and validating alternative substrates with lower environmental impact, safer handling, and similar or improved agronomic performance is critical for achieving greater sustainability in greenhouse vegetable production. Furthermore, substrate composition during nursery-level production influences early plant growth and root system development, thereby affecting crop productivity in subsequent cultivation stages [8].

Given these pressing environmental and agronomic challenges, this study aimed to (experiment 1) evaluate coconut fiber mixed with different proportions of perlite during seed germination and early growth as an environmentally sustainable alternative to rock wool and subsequently, (experiment 2) assess eco-physiological responses and nutrient uptake of lemon basil cultivated in hydroponic versus decoupled aquaponic systems, utilizing the best-performing coconut fiber-perlite mixture identified in the first phase along with rock wool as substrates.

By implementing soilless cultivation systems that rely on environmentally sustainable substrates and nutrient solutions, this research aims to developing more sustainable techniques for intensive vegetable production, thereby reducing synthetic fertilizer use and substrate-related environmental impacts.

## 2. Results

### 2.1. Experiment 1

#### 2.1.1. Seed Germination and Seedling Growth

Basil seed germination percentage was significantly higher in the treatment F70:P30 and the control (RW) (Table 1). Germination in the F70:P30% substrate was 26.1% points higher than in the F50:P50 treatment and 20.8% points higher than in the F90:P10 treatment.

The number of leaves per seedling was also significantly higher in all three treatments compared to the RW (Table 2). The F70:P30 treatment had the highest number of leaves, exceeding the control by 26.9%, followed by the F50:P50% and F90:P10 treatments, with an average leaf number of 22.9% higher than the control.

Plant height was also greater in all three treatments, averaging 7.24 cm, which was 24.2% taller than the control.

The total leaf area showed significant differences among the treatments. In particular, compared to the control, it was 44.6% greater in the F70:P30 treatment, 45.9% greater in the F50:P50 treatment, and 23% greater in the F90-P10 treatment.

The dry matter percentage in stems was significantly higher in the control treatment (RW), showing an increase of 22.9% compared to the substrate mix treatments. Similarly, the dry matter percentage in roots was higher in the RW, with an increment of 46.0% compared to the substrate mix treatments.

#### 2.1.2. Cluster Heatmap Analysis

**Figure 1:**
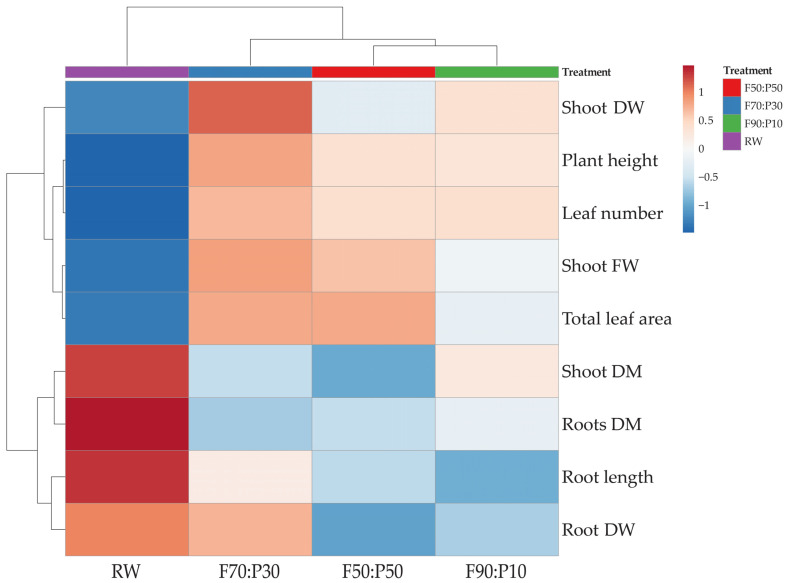
Cluster heatmap analysis displaying the relationships among growth parameters of lemon basil (*Ocimum × citriodorum*) seedlings grown on four different substrates: coconut fiber mixed with perlite at three ratios (F50:P50, F70:P30, F90:P10) and 100% rock wool (RW), in controlled growth chamber conditions. Each row represents a specific growth parameter (e.g., germination percentage, leaf number, root length), while each column represents an individual substrate treatment. Colors represent standardized values (ln(x + 1) transformed): higher values in red indicate above-average performance, whereas lower values in blue represent below-average performance. Hierarchical clustering was performed using Euclidean distances and complete linkage to group similar treatments and parameters together.

From the observation of the heatmap (x-axis, Figure 1), the clustering of the treatments reveals that RW formed a distinct cluster. Plants grown with RW exhibited a higher germination percentage, as well as increased dry matter content in both shoots and roots. In contrast, the Coconut-Perlite treatment formed a separate cluster, with the F70:P30 treatment showing, in order of proximity, the highest germination percentage compared to the other two treatments, along with longer roots and greater dry weight. Additionally, the F50:P50 and F90:P10 treatments generated a subcluster primarily characterized by lower germination activity and reduced root development.

### 2.2. Experiment 2

#### 2.2.1. Plant Growth

Basil plants exhibited similar leaf numbers, total leaf area, and total fresh biomass across all substrate treatments and cultivation systems (Table 3). The average leaf fresh weight was 50.8 g, and the mean number of leaves per plant was 129.6 n plant^−1^. The dry weight of the roots was 22.8% higher in plants grown on RW than in those grown on F. Compared to the hydroponic system, the percentage of dry matter in the leaves was 10% higher in the aquaponic cultivation system. Furthermore, compared to hydroponic cultivation, plants grown in aquaponic systems exhibited a 19% reduction in root length (Table 4).

#### 2.2.2. Gas Exchanges and Chl a Fluorescence Emission

Gas exchanges and *chlorophyll* fluorescence measurement showed no significant differences between the cultivation system and the growth on different substrates (Table 5). The mean net photosynthesis rate (A) was 8.51 µmol CO_2_ m^−2^ s^−1^ in hydroponic and 8.97 µmol CO_2_ m^−2^ s^−1^ in aquaponics systems. The maximum photochemical efficiency of the PSII (F_v_/F_m_) was 0.83 in the hydroponic system and 0.84 µmol m^−2^ s^−1^ in the aquaponics system. The actual yield of PSII (Φ_PSII_) and the non-photochemical quenching (NPQ) had mean values of 0.54 and 1.31, respectively, across all treatments. Similarly, the intrinsic water use efficiency (WUE_i_) showed no significant variation between treatments, with a mean value of 105.1 and 117.6 µmol CO_2_ m^−2^ s^−1^/mol H_2_O m^−2^ s^−1^ in hydroponic and aquaponic systems, respectively.

#### 2.2.3. Leaf Photosynthetic Pigments

The flavonoid index (FlvM) exhibited a significant difference among the cultivation systems, with a 52.5% increase observed in the aquaponic system compared to the hydroponic system (Table 6). In contrast, no significant differences were detected in the chlorophyll content index (CCla), which exhibited a mean index value of 23.8 for hydroponic and 21.6 for aquaponics systems.

#### 2.2.4. Mineral Content (Leaf, Stem, and Root)

The cultivation system and substrate did not influence nitrate (NO_3_) concentrations and mineral uptake in the leaves (Table 7). For the stems, compared to hydroponic cultivation, the aquaponic system resulted in a 21.9% higher calcium uptake. Similarly, with hydroponic cultivation, the magnesium uptake in the stems of plants grown in the aquaponic system was 37.5% higher. Regarding the interaction between the cultivation system and substrate, compared to both hydroponic cultivation and RW, the highest magnesium uptake in the stems was recorded in plants grown in the aquaponic system on coconut fiber substrate (AF) (Table 8). For the roots, compared to RW, calcium uptake was 20.8% higher in plants grown on F. Additionally, compared to the hydroponic system, magnesium uptake in the roots was 37.9% higher in plants grown in the aquaponic system (Table 9).

#### 2.2.5. Cluster Heatmap Analysis

**Figure 2:**
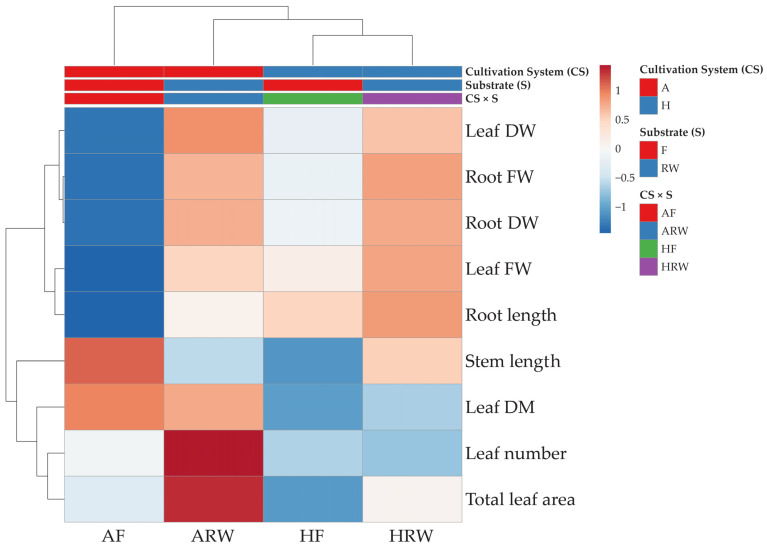
Cluster heatmap analysis illustrating morpho-anatomical parameters of lemon basil (*Ocimum × citriodorum*) plants grown on two different substrates: 70% coconut fiber mixed with 30% perlite (F) and 100% rock wool (RW) and in two different cultivation systems, hydroponic (H) or aquaponic (A). Rows represent the measured parameters (e.g., leaf number, total leaf area, root length, flavonoid index), and columns represent each combination of substrate and cultivation system. Color intensity reflects standardized values (ln(x + 1) transformed), with red indicating higher-than-average values and blue indicating lower-than-average values. Hierarchical clustering was conducted using Euclidean distances and complete linkage.

To obtain an in-depth overview of the morpho-anatomical and eco-physiological variations induced by the two cultivation systems and growth substrates, a clustered heatmap was created for all the parameters to highlight these differences better (Figure 2). As a result, the primary factor influencing clustering was the cultivation system rather than the substrates. Plants grown in the aquaponic system, particularly when combined with F substrate, demonstrated significantly increased stem length and leaf dry matter. Additionally, the aquaponic system showed a markedly higher leaf number and total leaf area, especially when associated with RW substrate. Furthermore, the fresh and dry weights of leaves and roots were notably higher in the aquaponic system when associated with RW substrate. In contrast, the hydroponic cultivation system, regardless of the substrate on which the plants were grown, yielded lower values for leaf number, total leaf area, and leaf dry matter than the aquaponic system. Nevertheless, the hydroponic system produced results comparable to those of the aquaponic system concerning both fresh and dry weights of leaves and roots, as well as root length, particularly when plants were grown on RW substrate.

## 3. Discussion

This study evaluated the potential of coconut fiber substrates combined with perlite as sustainable alternatives to rock wool (experiment 1) and compared plant growth, nutrient uptake, and eco-physiological responses under hydroponic and aquaponic conditions (experiment 2) of lemon basil (*Ocimum × citriodorum*). Our key findings from experiment 1 revealed that a substrate mix composed of 70% coconut fiber and 30% perlite (F70:P30) promoted optimal seed germination and seedling growth compared to other tested substrates and was comparable or superior to rock wool. Furthermore, in experiment 2, while total leaf biomass did not differ significantly between hydroponic and aquaponic systems, aquaponics increased leaf dry matter and flavonoid content and improved calcium and magnesium uptake compared to hydroponics, indicating potential nutritional and phytochemical advantages. These findings align with previous research indicating the potential of coconut fiber substrates and aquaponics to enhance plant health and sustainability [12,13].

Environmental challenges linked to intensive agriculture practices in soil-grown leafy vegetables, such as heavy metal contamination, have become increasingly concerning in southern Italy, especially in the Campania region [3]. A more sustainable and safer alternative is represented by the adoption of closed-loop soilless cultivation systems. Soilless cultivation, such as hydroponics and aquaponics, represents viable solutions because it reduces plant exposure to contaminated soils, minimizes agrochemical use, and significantly improves resource-use efficiency compared to traditional soil-based cultivation practices [14,15].

Traditionally, rock wool (RW) has been widely used in commercial greenhouse production due to its favorable physical properties for root development [16]. However, RW is environmentally problematic, expensive to dispose of, and hazardous to human health during handling [11].

Our results highlight coconut fiber substrates, particularly the F70:P30 blend, as a promising alternative substrate. In alignment with studies, the improved water retention and high cation exchange capacity (CEC) of coconut fiber likely facilitated superior germination and early seedling growth [17]. Additionally, the enhanced growth (leaf number, plant height, leaf area) observed in seedlings grown on F70:P30 indicates that this substrate might have provided optimal conditions for early establishment, potentially influencing subsequent plant productivity positively.

In Experiment 2, which focuses on the comparison of aquaponic and hydroponic systems, despite the initial differences observed during germination and seedling stages (experiment 1), the total fresh biomass and leaf number did not significantly differ among treatments. This outcome supports previous research that reported comparable yields between hydroponic and aquaponic systems [12]. Interestingly, root dry biomass was greater in RW-grown plants, likely due to RW’s stable physical structure enhancing root growth [18]. Nevertheless, from an environmental sustainability perspective, coconut fiber substrates offer clear advantages over RW, with lower environmental impact, safer handling, and reduced disposal costs [11,16].

While hydroponics remains a widely adopted soilless technique, its dependence on synthetic fertilizers raises concerns regarding environmental sustainability [15]. In contrast, decoupled aquaponics offers a promising alternative by utilizing nutrient-rich aquaculture water, thereby significantly reducing the need for supplementary fertilization [19,20]. Aquaponic cultivation resulted in a 10% higher leaf dry matter content compared to hydroponics. This finding aligns with recent hypotheses suggesting beneficial interactions between aquaponic solution microbial communities and plant roots, potentially increasing nutrient availability and promoting physiological adaptations [21,22]. Moreover, the significantly higher flavonoid content (52.5% increase) observed in aquaponically grown lemon basil plants highlights aquaponics’ potential to enhance phytochemical quality. Previous studies have also reported increased flavonoid concentrations in aromatic plants grown aquaponically [23,24]. This increase in flavonoids may be linked to osmotic stress resulting from higher sodium concentrations in aquaponic solutions, prompting plants to activate secondary metabolic pathways as a protective mechanism against oxidative stress [25,26,27]. Lemon basil (*Ocimum × citriodorum*), specifically, appears particularly responsive to mild salinity stress, producing elevated flavonoid concentrations under such conditions [28]. Root length was significantly shorter in aquaponic-grown plants compared to hydroponics, likely due to increased sodium and salinity stress [25]. However, this reduced root growth did not negatively affect overall biomass production or photosynthetic performance. Indeed, gas exchange and chlorophyll fluorescence measurements (net photosynthesis, PSII efficiency) showed no significant differences between systems or substrates, in line with previous findings indicating stable photosynthetic performance under both cultivation methods [12,29]. Regarding mineral nutrition, aquaponically cultivated plants exhibited significantly higher calcium and magnesium uptake in stems and roots compared to hydroponically grown plants. This result supports previous findings demonstrating distinct nutrient uptake patterns influenced by microbial activities inherent to aquaponic nutrient solutions [30]. The microbial diversity within aquaponic systems, particularly nitrifying bacteria, can enhance nutrient solubility and uptake efficiency, explaining these observed differences [31,32]. Additionally, the chemical properties of coconut fiber substrates, particularly their high cation exchange capacity (CEC), likely enhanced nutrient availability to the plants [33]. Probably, RW, due to its favorable physical properties, promoted root growth; however, as an inert substrate, it did not enhance nutrient uptake to the same as coconut fiber. Although plants grown in RW developed more root biomass, this did not translate into significant differences in nutrient composition compared to those grown on F, as the high CEC allowed plants with less root system to absorb comparable amounts of nutrients as those grown on RW. However, further research is needed to clarify the precise interactions between coconut fiber substrates, aquaponic microbial communities, and plant nutrient uptake dynamics. Despite being a decoupled system, the aquaponic setup still contains residues of fish feed and fish feces, which inevitably influence the nutrient solution composition over time [26]. Although pH, CEC, and other parameters were carefully managed to remain similar between aquaponic and hydroponic solutions, the presence of these organic inputs leads to ongoing changes in nutrient availability and solution dynamics. This aspect represents a key difference from hydroponic systems and highlights the complex interactions between microbial communities and nutrient cycling in aquaponics. Such considerations suggest valuable directions for future research on optimizing nutrient solution characteristics concerning fish feeding regimes in aquaponic cultivation.

## 4. Materials and Methods

### 4.1. Experiment 1: Seed Germination and Seedling Growth

#### 4.1.1. Experimental Site and Duration

The germination experiment was conducted from 15 November to 6 December 2022 at the University Centre for Innovation and Development in the Food Industry (CAISIAL) of the University of Naples Federico II—Department of Agriculture, University of Naples Federico II, Portici, Italy (40°48′57.9″ N 14°21′01.6″ E, 29 m a.s.l.), within a climate-controlled chamber.

#### 4.1.2. Plant Material and Experimental Conditions

Seeds of lemon basil (*Ocimum × citriodorum*) (Bingenheimer Saatgut, Echzell, Germany) were used. The experiment was conducted under controlled conditions: ambient temperature was set at 26 °C, while inside the germination containers, temperature was maintained at 29 °C, with relative humidity (R.H.) kept constant at 89%. Seeds were initially hydrated with a half-strength Hoagland nutrient solution, with a pH of 6.2 and electrical conductivity (EC) of 1600 µS/cm.

#### 4.1.3. Substrates and Sowing Procedure

Four substrate treatments were tested: (1) 100% rock wool (RW; control), (2) 50% coconut fiber (F) mixed with 50% perlite (P) (F50:P50), (3) 70% coconut fiber mixed with 30% perlite (F70:P30), and (4) 90% coconut fiber mixed with 10% perlite (F90:P10). A completely randomized design was adopted, with three replicate containers per substrate type. Each container contained 12 sowing holes, with two seeds sown per hole (24 seeds per container, 72 seeds per treatment). The sowing holes measured 3.81 × 3.81 cm with a depth of 0.5 cm, resulting in a soil volume of 7.25 cm^3^ per hole for both substrates.

#### 4.1.4. Germination and Seedling Management

Seeds underwent an initial 48-h dark period, followed by illumination at 243 µmol m^−2^ s^−1^ photosynthetic photon flux density (PPFD) provided by full-spectrum fluorescent lamps (ATI Powermodule, Viareggio, LU, Italy) positioned 50 cm above seedling containers. After the first 7 days, light intensity was increased to 343 µmol m^−2^ s^−1^ with a 16/8 h photoperiod (day/night). Initially, substrates were hydrated every two days with osmotic water until the emergence of the first radicle. Subsequently, substrates were irrigated at the same frequency with half-strength Hoagland nutrient solution (EC 1100 µS/cm; pH 5.7), ensuring consistent moisture without water stagnation.

#### 4.1.5. Seedling Growth Measurements

Seed germination was monitored daily over 7 consecutive days. A seed was considered germinated upon emergence of the two cotyledons above the substrate surface. The germination percentage (% GP) was calculated on day 7 as (number of germinated seeds/total number of seeds sown) × 100. At twenty days after sowing, seedling height (collar base to vegetative apex), leaf number, total leaf area, and root length were measured by analyzing digital images using ImageJ software, version 1.50i (Wayne Rasband National Institute of Health, Bethesda, MD, USA). The fresh weight of the shoot and root was measured using an electronic balance; dry weights were determined after oven drying at 70 °C for 48 h.

### 4.2. Experiment 2: Comparison of Aquaponic and Hydroponic Systems

#### 4.2.1. Experimental Site, Duration, and Setup

This greenhouse experiment was conducted from 6 December 2022 to 13 January 2023 at the same university facility (40°48′57.9″ N, 14°21′01.6″ E). Twenty-day-old lemon basil seedlings grown on 70% coconut fiber mixed with 30% perlite (F) and on rock wool (RW) substrates (Experiment 1) were transplanted into floating raft systems of decoupled aquaponic (A) and hydroponic (H) units at a density of 20 plants m^−2^. These two treatments were selected based on the results of the germination experiment: RW served as the control substrate, while F70:P30 achieved the highest germination percentage, outperforming F50:P50 and F90:P10 by 26.1 and 20.8 percentage points, respectively.

#### 4.2.2. Greenhouse and Cultivation Systems Description

The experiment utilized a recirculating aquaponic system (RAS) prototype comprising four aquaculture tanks (2800 L each). Nile tilapia (*Oreochromis niloticus*) was stocked at an average density of 8.7 ± 5.4 kg m^−3^. The RAS included an 800-L mechanical and biological filtration unit (Superbead, Air-aqua, Staphorst, The Netherlands), a 400-L trickling filter (Scubla srl, Udine, Italy), and a 40-W UV sterilizer (Air-aqua, Staphorst, The Netherlands). Air was supplied at 0.05 v v^−1^ min^−1^. Floating raft units (2 m^2^ each) were independently managed as decoupled systems for aquaponic and hydroponic treatments.

#### 4.2.3. Decoupled Aquaponic and Hydroponic Nutrient Solution Management

Initially, the aquaponic tank was filled with RAS water and subsequently decoupled. Both systems were maintained at a constant water temperature of 23 °C, with daily monitoring of pH and EC using Thermo Scientific Expert Testers (Segrate, Italy). Nutrient solutions were analyzed biweekly, and the pH was adjusted to a target of 6.2–6.3 using nitric acid (HNO_3_) and potassium hydroxide (KOH). The electrical conductivity (EC) was maintained at approximately 1600 µS cm^−1^. The half-strength Hoagland hydroponic nutrient solution was prepared by dissolving the following salts per liter of solution: KNO_3_ = 0.75 g L^−1^; CaCl_2_ = 0.28 g L^−1^; MgSO^4^ * 7H_2_O = 0.25 g L^−1^; H_3_PO^4^= 0.04 g L^−1^; FeSO^4^ * 7H_2_O = 0.02 g L^−1^; Fe-EDTA = 0.04 g L^−1^, H_3_BO_3_ (0.0014 g L^−1^), MnCl_2_·4H_2_O (0.0011 g L^−1^), ZnSO_4_·7H_2_O (0.0001 g L^−1^), CuSO_4_·5H_2_O (0.00004 g L^−1^), and H_2_MoO_4_·H_2_O (0.0001 g L^−1^)”. Nutrient concentrations for both systems are detailed in Table 10.

#### 4.2.4. Plant Growth Measurements

At 38 days after transplanting (DAT), corresponding to 59 days after sowing, plant height, leaf number, total leaf area, and root length were measured using ImageJ software, version 1.50i (Wayne Rasband, National Institute of Health, Bethesda, MD, USA). Fresh and dry weights of shoots and roots were recorded after drying samples at 70° C for 48 h.

#### 4.2.5. Leaf Vegetation Indices

Leaf anthocyanin (AnthM), flavonoid (FlvM), and chlorophyll indices (CCla) were measured at 38 DAT using a portable MPM-100, Multi Pigment Meter (ADC BioScientific Ltd., Hoddesdon, UK). The anthocyanin index was calculated at 660 and 525 nm as AnthM = log (f_660_/f _525_). The flavonoid index was calculated at 660 and 375 nm as FlvM = log (f_660_/f_375_). The chlorophyll content index (CCIa) was calculated using the formula CCIa = (T_850_/T_720_) × 0.86532 + 0.39, where T_850_ and T_720_ are the transmittance measurements at 850 nm and 720 nm, respectively.

#### 4.2.6. Gas Exchanges and Chlorophyll Fluorescence Measurements

Leaf gas exchange was measured at 38 DAT on one fully expanded leaf from each plant on 3 plants × 3 replicates × cultivation system × substrate, using an Infrared Gas Analyser (LCi T, ADC Bioscientific Ltd., Hoddesdon, UK). The measurements were conducted at noon under ambient CO_2_ (on average 513 ± 25 ppm), mean temperature 28 °C, R.H. 33%, and PPFD 486 µmol m^−2^ s^−1^. Intrinsic water use efficiency (WUE_i_) was calculated as leaf net photosynthesis (A) divided by stomatal conductance (g_s_).

On the same leaves used for gas exchange measurements, the evaluation of chlorophyll fluorescence was performed. A portable Plant Stress Kit (Opti-Sciences Inc., Hudson, NY, USA) was utilized, and light-induced measurements were conducted using a ΦPSII meter, applying a saturating pulse of 4286 µmol m^−2^ s^−1^ for 1.1 s to obtain the maximum light-adapted fluorescence (F_m_) and steady-state fluorescence (F_s_). For dark-adapted measurements, the leaves were dark-adapted for 30 min with a dark leaf clip (Opti-Sciences Inc., Hudson, NY, USA). An F_v_/F_m_ meter (Opti-Sciences Inc., Hudson, NY, USA), capable of delivering a 1.0-s saturating light pulse (3429 µmol m^−2^ s^−1^), was employed to obtain the F_m_ and F_0_ values. The PSII maximal photochemical efficiency (F_v_/F_m_) was determined using F_v_/F_m_ = (F_m_ − F_0_)/F_m_. The quantum yield of PSII electron transport (Φ_PSII_) was computed as Φ_PSII_ = (F_m’_ − F_s_)/F_m’_ [34]. The non-photochemical quenching (NPQ) was calculated as (F_m_/F_m’_) − 1 [35].

#### 4.2.7. Leaf, Stem, and Root Mineral Content Analyses

Mineral content (NO_3_^−^, P, K, Ca, Mg) of leaves, stems, and roots were analyzed at 38 DAT using 38 DAT on samples from each of 3 plants × 3 replicates × substrate × cultivation system. A 250 mg aliquot of a ground dry tissue sample was utilized for the determination of leaf mineral composition according to the procedures outlined by Pannico et al. [36]. Subsequently, mineral analysis was conducted following 0.45-micrometer filtration using an ion chromatography system (model ICS-3000, Dionex, Sunnyvale, CA, USA), with quantification facilitated by an electrical conductivity detector equipped with IonPac CS12A and IonPac AS11-HC analytical columns for cationic and anionic content analysis, respectively (Dionex, Sunnyvale, CA, USA). Accounting for the dry matter content of the leaves, all mineral (leaf, stem, and root) uptake values were calculated by multiplying the concentrations expressed in grams per kilogram (g kg^−1^) on a dry weight (DW) basis by the dry weight of the analyzed organ and expressed as gram per plant (g plant^−1^). Only nitrate was expressed as concentration (g kg^−1^) on a fresh weight (FW) basis.

#### 4.2.8. Statistical Analysis

*Experiment 1*

Measurements and statistical analysis were carried out on 4 plants × 3 replicates × 4 substrates, resulting in 48 plants. Data were analyzed using one-way ANOVA. The analysis of the variance was performed using SPSS software version 27 (IBM, Chicago, IL, USA). Means were compared by Tukey’s HSD post hoc test at a significance level of *p* < 0.05. A cluster heatmap was generated with ClustVis online software [37], using Euclidean distance and complete linkage clustering; data were log(x + 1) transformed.

*Experiment 2*

Measurements and statistical analysis were carried out on 3 plants × 3 replicates × 2 substrates × 2 cultivation systems, resulting in 36 plants. Data were analyzed using two-way ANOVA. The analysis of the variance was performed using SPSS software version 27 (IBM, Chicago, IL, USA). Means were compared by Tukey’s HSD post hoc test at a significance level of *p *< 0.005. A cluster heatmap was generated with ClustVis online software [37], using Euclidean distance and complete linkage clustering; data were log(x + 1) transformed.

## 5. Conclusions

This research highlights the effectiveness of the F70:P30 coconut fiber-perlite substrate mix in optimizing seed germination and early plant growth, providing a sustainable and environmentally friendly alternative to rock wool substrate. This alternative reduces disposal costs and minimizes potential health risks associated with rock wool handling.

Additionally, decoupled aquaponics proved to be a viable cultivation method, achieving yields comparable to traditional hydroponics while significantly decreasing dependence on chemical fertilizers.

The observed increase in flavonoid content in aquaponics-grown lemon basil suggests additional nutritional and nutraceutical benefits, potentially attributable to mild osmotic stress conditions.

This enhancement offers growers a marketing advantage and the potential for higher economic returns from health-conscious consumers.

Collectively, these findings offer direct practical implications for commercial greenhouse production, supporting the adoption of more sustainable, cost-effective, and resource-efficient agricultural practices. Future research should further investigate the integration and optimization of sustainable substrates and aquaponic systems, particularly for commercial vegetable production in regions affected by soil contamination.

## Figures and Tables

**Table 1:** Percentage germination of seeds, total shoot Dry Matter (DM), total root Dry Matter (DM), total plant height, total root length of seedlings of Lemon basil (*Ocimum × citriodorum*) cultivated on different substrates under controlled growth chamber conditions. The substrates compared were: 50% coconut fiber mixed with 50% perlite (F50:P50), 70% coconut fiber mixed with 30% perlite (F70:P30), 90% coconut fiber mixed with 10% perlite (F90:P10), and 100% rock wool (RW; control).

Treatment	Germination	Shoot DM	Root DM	Plant Height	Root Length
Substrate	%			cm Plant^−1^	
F50:P50	63.89 ± 3.67 b	8.41 ± 0.32 b	4.43 ± 0.08 b	7.12 ± 0.28 a	13.67 ± 0.91
F70:P30	80.56 ± 5.55 a	8.86 ± 0.23 b	4.30 ± 0.1 b	7.47 ± 0.31 a	14.71 ± 1.51
F90:P10	66.67 ± 2.41 b	9.79 ± 0.15 ab	4.75 ± 0.05 b	7.13 ± 0.35 a	13.08 ± 0.9
RW 100%	81.94 ± 5.55 a	11.09 ± 0.46 a	6.57 ± 0.73 a	5.83 ± 0.06 b	16.17 ± 1.18
*Significance*	0.04 *	0.00 **	0.01 **	0.01 *	0.32 ^ns^

Data represent means ± standard error (n = 12). Different letters indicate significant differences among treatments according to Tukey’s HSD post hoc test. Significance levels: ns = not significant; * *p* < 0.05, ** *p* < 0.01, *** *p* < 0.001.

**Table 2:** Total shoot Fresh Weight (FW), total shoot Dry Weight (DW), total leaf number, and total leaf area of Lemon basil (*Ocimum × citriodorum*) seedlings cultivated on different substrates under controlled growth chamber conditions. The substrates compared were: 50% coconut fiber mixed with 50% perlite (F50:P50), 70% coconut fiber mixed with 30% perlite (F70:P30), 90% coconut fiber mixed with 10% perlite (F90:P10), and 100% rock wool (RW; control).

Treatment	Shoot FW	Shoot DW	Leaf Number	Total Leaf Area
Substrate	g Plant^−1^		n Plant^−1^	cm^2^ Plant^−1^
F50:P50	1.09 ± 0.08 a	0.10 ± 0.01	7.58 ± 0.22 a	24.78 ± 2.10 a
F70:P30	1.13 ± 0.07 a	0.10 ± 0.01	7.83 ± 0.2 a	24.55 ± 0.81 a
F90:P10	0.97 ± 0.05 ab	0.10 ± 0.01	7.58 ± 0.17 a	20.89 ± 0.66 ab
RW 100%	0.78 ± 0.02 b	0.09 ± 0.00	6.17 ± 0.17 b	16.98 ± 0.39 b
*Significance*	0.01 *	0.50 ^ns^	0.00 **	0.01 *

Data represent means ± standard error (n = 12). Different letters indicate significant differences among treatments according to Tukey’s HSD test. Significance levels: ns = not significant; * *p* < 0.05, ** *p* < 0.01, *** *p* < 0.001.

**Table 3:** Total leaf number, total leaf area, total Fresh Weight (FW), total Dry Weight (DW), total root Fresh Weight (FW) of Lemon basil (*Ocimum × citriodorum*) plants cultivated on two different substrates 70% coconut fiber mixed with 30% perlite (F) and 100% rock wool (RW) and in two different cultivation systems: aquaponics (A) and hydroponics (H).

Treatments		Leaf Number	Leaf FW	Leaf DW	Root FW	Total Leaf Area
Cultivation System (CS)	Substrate (S)	(n Plant^−1^)	(g Plant^−1^)			(cm^2^ Plant^−1^)
A	F	127.55 ± 7.29	40.56 ± 4.36	3.81 ± 0.38	26.33 ± 2.79	1098.02 ± 78.09
	RW	143.77 ± 13.66	53.22 ± 4.86	5.02 ± 0.49	33.56 ± 2.94	1307.38 ± 103.30
Mean		135.67	46.89	4.42	29.94	1202.70
H	F	125.44 ± 12.10	53.67 ± 8.06	4.64 ± 0.76	31.11 ± 3.66	1060.47 ± 135.46
	RW	121.77 ± 6.59	55.78 ± 5.21	4.85 ± 0.48	34.67 ± 3.57	1147.14 ± 92.41
Mean		123.61	54.72	4.75	32.89	1103.81
**Significance**						
Cultivation system (CS)		0.25 ^ns^	0.18 ^ns^	0.56 ^ns^	0.37 ^ns^	0.35 ^ns^
Substrates (S)		0.55 ^ns^	0.21 ^ns^	0.20 ^ns^	0.11 ^ns^	0.17 ^ns^
CS × S		0.46 ^ns^	0.25 ^ns^	0.42 ^ns^	0.29 ^ns^	0.37 ^ns^

Data represent means ± standard error (n = 9). Different letters indicate significant differences among treatments according to Tukey’s HSD test. Significance levels: ns = not significant; * *p* < 0.05, ** *p* < 0.01, *** *p* < 0.001.

**Table 4:** Root dry weight (DW), leaf dry matter (DM) percentage, stem length, and root length of Lemon basil (*Ocimum × citriodorum*) plants cultivated on two different substrates: 70% coconut fiber mixed with 30% perlite (F) and 100% rock wool (RW) and in two different cultivation systems: aquaponics (A) and hydroponics (H).

Treatments		Root DW	Leaf DM	Stem Length		Root Length
Cultivation System (CS)	Substrate (S)	(g Plant^−1^)	(%)		(cm Plant^−1^)	
A	F	1.71 ± 0.14	9.49 ± 0.22 a	34.36 ± 2.57		13.704 ± 2.16
	RW	2.31 ± 0.18	9.40 ± 0.28 a	31.73 ± 2.45		15.46 ± 0.88
Mean		2.01	9.45	33.05		14.58
H	F	2.06 ± 0.18	8.51 ± 0.31 b	30.80 ± 2.08		17.42 ± 1.68
	RW	2.32 ± 0.19	8.68 ± 0.25 b	32.93 ± 1.62		18.57 ± 1.33
Mean		2.19	8.59	31.87		18.00
**Significance**						
Cultivation system (CS)		0.31 ^ns^	0.03 *	0.59 ^ns^		0.04 *
Substrates (S)		0.02 *	0.88 ^ns^	0.91 ^ns^		0.37 ^ns^
CS × S		0.06 ^ns^	0.03 *	0.69 ^ns^		0.16 ^ns^

Data represent means ± standard error (n = 9). Different letters indicate significant differences among treatments according to Tukey’s HSD test. Significance levels: ns = not significant; * *p* < 0.05, ** *p* < 0.01, *** *p* < 0.001.

**Table 5:** Gas exchange and chlorophyll fluorescence parameters of lemon basil (*Ocimum × citriodorum*) plants grown on two different substrates: 70% coconut fiber mixed with 30% perlite (F) and 100% rock wool (RW) and in two different cultivation systems hydroponic (H) or aquaponic (A). Parameters measured include leaf net photosynthesis rate (A), intrinsic water use efficiency (WUE_i_), quantum yield of photosystem II (Φ_PSII_), maximal photochemical efficiency of photosystem II (F_v_/F_m_), and non-photochemical quenching (NPQ).

Treatments		A	WUE_i_	Φ_PSII_	F_v_/F_m_	NPQ
Cultivation System (CS)	Substrate (S)	(µmol CO_2_ m^−2^ s^−1^)	(µmol CO_2_ m^−2^ s^−1^/mol H_2_O m^−2^ s^−1^)			
A	F	9.27 ± 0.89	107.67 ± 10.97	0.62 ± 0.04	0.84 ± 0.00	1.07 ± 0.09
	RW	8.67 ± 0.86	127.53 ± 14.44	0.49 ± 0.07	0.83 ± 0.01	1.48 ± 0.21
Mean		8.97	117.6	0.55	0.84	1.28
H	F	8.24 ± 0.48	112.56 ± 8.55	0.49 ± 0.06	0.83 ± 0.01	1.31 ± 0.14
	RW	8.78 ± 1.02	97.56 ± 12.05	0.57 ± 0.05	0.83 ± 0.01	1.36 ± 0.12
Mean		8.51	105.06	0.53	0.83	1.34
**Significance**						
Cultivation system (CS)		0.58 ^ns^	0.30 ^ns^	0.72 ^ns^	0.82 ^ns^	0.68 ^ns^
Substrates (S)		0.97 ^ns^	0.84 ^ns^	0.67 ^ns^	0.87 ^ns^	0.14 ^ns^
CS × S		0.50 ^ns^	0.15 ^ns^	0.07 ^ns^	0.96 ^ns^	0.24 ^ns^

Data represent means ± standard error (n = 9). Different letters indicate significant differences among treatments according to Tukey’s HSD test. Significance levels: ns = not significant; * *p* < 0.05, ** *p* < 0.01, *** *p* < 0.001.

**Table 6:** Flavonoid index (FlvM) and chlorophyll content index (CCla) of lemon basil (*Ocimum × citriodorum*) plants grown on two different substrates: 70% coconut fiber mixed with 30% perlite (F) and 100% rock wool (RW) and in two different cultivation systems hydroponic (H) or aquaponic (A).

Treatments		FlvM	CCla
Cultivation System (CS)	Substrate (S)	Index	
A	F	0.92 ± 0.08	21.54 ± 1.33
	RW	0.93 ± 0.03	21.64 ± 2.23
Mean		0.93	21.60
H	F	0.66 ± 0.09	22.89 ± 2.00
	RW	0.56 ± 0.03	24.6 ± 200
Mean		0.61	23.75
**Significance**			
Cultivation system (CS)		0.00 ***	0.27 ^ns^
Substrates (S)		0.51 ^ns^	0.64 ^ns^
CS × S		0.4 ^ns^	0.68 ^ns^

Data represent means ± standard error (n = 9). Different letters indicate significant differences among treatments according to Tukey’s HSD test. Significance levels: ns = not significant; * *p* < 0.05, ** *p* < 0.01, *** *p* < 0.001.

**Table 7:** Leaf nitrate (NO_3_^−^) concentrations and phosphorus (P), potassium (K), calcium (Ca), magnesium (Mg), and sulfur (S) uptake of lemon basil (*Ocimum × citriodorum*) plants grown on two different substrates: 70% coconut fiber mixed with 30% perlite (F) and 100% rock wool (RW) and in two different cultivation systems hydroponic (H) or aquaponic (A). Minerals are expressed as follows: nitrate (NO_3_^−^) in g kg^−1^ fresh weight (f.w.), all other nutrients as g plant^−1^ dry weight (d.w.).

Treatments		NO_3_	P	K	Ca	Mg	S
Cultivation System (CS)	Substrate (S)	mg kg^−1^ f.w.	g Plant^−1^ d.w.				
A	F	2451.67 ± 631.40	17.67 ± 1.67	153.67 ± 13.22	63.67 ± 3.84	18.67 ± 1.45	7.33 ± 1.20
	RW	5457.67 ± 208.73	23.00 ± 2.00	181.67 ± 24.18	82.00 ± 7.00	25.00 ± 1.53	18.67 ± 1.76
Mean		3954.67	20.34	167.67	72.83	21.83	13.00
H	F	3402.00 ± 1480.88	18.33 ± 9.24	211.00 ± 48.40	59.00 ± 12.74	18.00 ± 4.04	17.33 ± 5.04
	RW	1643.33 ± 815.76	16.67 ± 6.98	224.67 ± 10.81	66.67 ± 2.85	19.33 ± 0.67	20.33 ± 14.44
Mean		2522.67	17.50	217.84	62.83	18.67	18.83
**Significance**							
Cultivation system (CS)		0.154 ^ns^	0.646 ^ns^	0.115 ^ns^	0.228 ^ns^	0.206 ^ns^	0.472 ^ns^
Substrates (S)		0.512 ^ns^	0.765 ^ns^	0.484 ^ns^	0.128 ^ns^	0.135 ^ns^	0.380 ^ns^
CS × S		0.08 ^ns^	0.877 ^ns^	0.356 ^ns^	0.245 ^ns^	0.201 ^ns^	0.648 ^ns^

Data represent means ± standard error (n = 9). Different letters indicate significant differences among treatments according to Tukey’s HSD test. Significance levels: ns = not significant; * *p* < 0.05, ** *p* < 0.01, *** *p* < 0.001.

**Table 8:** Stem nitrate (NO_3_^−^) concentrations and phosphorus (P), potassium (K), calcium (Ca), and magnesium (Mg) uptake of lemon basil (*Ocimum × citriodorum*) plants grown on two different substrates: 70% coconut fiber mixed with 30% perlite (F) and 100% rock wool (RW) and in two different cultivation systems hydroponic (H) or aquaponic (A). Minerals are expressed as follows: nitrate (NO_3_^−^) in g kg^−1^ fresh weight (f.w.), all other nutrients as g plant^−1^ dry weight (d.w.).

Treatments		NO_3_	P	K	Ca	Mg
Cultivation System (CS)	Substrate(S)	mg kg^−1^ f.w.	g plant^−1^ d.w.			
A	F	2485.00 ± 694.26	2.00 ± 0.00	94.00 ± 3.06	7.00 ± 0.00	4.00 ± 0.00 a
	RW	5783.00 ± 3230.14	1.67 ± 0.33	102.00 ± 8.72	6.00 ± 0.58	3.33 ± 0.33 a
Mean		4134.00	1.83	98.00	6.50	3.67
H	F	3712.00 ± 452.27	1.67 ± 0.33	91.00 ± 12.12	5.33 ± 0.67	2.67 ± 0.33 b
	RW	2020.67 ± 397.76	1.67 ± 0.33	87.33 ± 3.18	5.33 ± 0.33	2.67 ± 0.33 b
Mean		2866.33	1.67	89.17	5.33	2.67
**Significance**						
Cultivation system (CS)		0.472 ^ns^	0.580 ^ns^	0.289 ^ns^	0.038 *	0.009 **
Substrates (S)		0.645 ^ns^	0.580 ^ns^	0.788 ^ns^	0.320 ^ns^	0.282 ^ns^
CS × S		0.44 ^ns^	0.802 ^ns^	0.609 ^ns^	0.109 ^ns^	0.032 *

Data represent means ± standard error (n = 9). Different letters indicate significant differences among treatments according to Tukey’s HSD test. Significance levels: ns = not significant; * *p* < 0.05, ** *p* < 0.01, *** *p* < 0.001.

**Table 9:** Root nitrate (NO_3_^−^) concentrations and phosphorus (P), potassium (K), calcium (Ca), magnesium (Mg) uptake of lemon basil (*Ocimum × citriodorum*) plants grown on two different substrates: 70% coconut fiber mixed with 30% perlite (F) and 100% rock wool (RW) and in two different cultivation systems hydroponic (H) or aquaponic (A). Minerals are expressed as follows: nitrate (NO_3_^−^) in g kg^−1^ fresh weight (f.w.), all other nutrients as g plant^−1^ dry weight (d.w.).

Treatments		NO_3_	P	K	Ca	Mg
Cultivation System (CS)	Substrate (S)	mg kg^−1^ f.w.	g Plant^−1^ d.w.			
A	F	2998.70 ± 63.19	0.58 ± 0.12	75.94 ± 6.77	4.28 ± 0.22	10.25 ± 0.63
	RW	2476.03 ± 158.60	0.68 ± 0.25	81.95 ± 10.90	3.17 ± 0.24	11.43 ± 1.69
Mean		2737.36	0.63	78.95	3.73	10.84
H	F	2356.53 ± 125.05	0.68 ± 0.10	77.77 ± 13.13	3.73 ± 0.07	7.70 ± 1.33
	RW	2491.60 ± 178.48	0.54 ± 0.18	85.25 ± 4.71	3.46 ± 0.37	8.04 ± 0.49
Mean		2424.07	0.61	81.51	3.60	7.86
**Significance**						
Cultivation system (CS)		0.053 ^ns^	0.915 ^ns^	0.793 ^ns^	0.620 ^ns^	0.032 *
Substrates (S)		0.199 ^ns^	0.920 ^ns^	0.497 ^ns^	0.020 *	0.527 ^ns^
CS × S		0.050 ^ns^	0.920 ^ns^	0.897 ^ns^	0.066 ^ns^	0.141 ^ns^

Data represent means ± standard error (n = 9). Different letters indicate significant differences among treatments according to Tukey’s HSD test. Significance levels: ns = not significant; * *p* < 0.05, ** *p* < 0.01, *** *p* < 0.001.

**Table 10:** Nutrient concentration (mg L^−1^) in hydroponic (H) and aquaponic (A) systems.

System	NO_3_	PO_4_	SO_4_	K	Ca	Mg	Na	Cl
H	390.28	45.70	80.43	95.77	113.64	35.98	10.35	7.63
A	469.93	50.04	60.04	40.65	107.46	31.77	41.23	59.08

## Data Availability

The data are contained within the article.
